# Successful remote treatment of a client with Hikikomori using internet-delivered cognitive therapy for social anxiety disorder: a case report

**DOI:** 10.3389/fpsyt.2024.1368722

**Published:** 2024-05-27

**Authors:** Motohiro Sakai, Naoki Yoshinaga, Graham R. Thew, David M. Clark

**Affiliations:** ^1^ Faculty of Education, University of Miyazaki, Miyazaki, Japan; ^2^ School of Nursing, Faculty of Medicine, University of Miyazaki, Miyazaki, Japan; ^3^ Oxford Health National Health Service Foundation Trust, Oxford, United Kingdom; ^4^ Department of Experimental Psychology, University of Oxford, Oxford, United Kingdom

**Keywords:** case study, cognitive therapy, hikikomori, internet-based intervention, social anxiety disorder

## Abstract

Hikikomori (prolonged social withdrawal) has been discussed as a hidden worldwide epidemic and a significant social and healthcare issue. Social anxiety disorder is the most common psychiatric disorder preceding the onset of Hikikomori. Although studies exist suggesting the effectiveness of family-support interventions, little is known about psychotherapeutic approaches for Hikikomori individuals. Here, we present a case of Hikikomori wherein an internet-delivered cognitive therapy for social anxiety disorder (iCT-SAD) worked effectively in improving the client’s social anxiety symptoms and social interaction behaviors. This case study demonstrates the principle that evidence-based psychological interventions focusing on social anxiety can be effective for clients with Hikikomori. Furthermore, the online mode of treatment delivery, along with a variety of relevant modules, may facilitate clients’ engagement with treatment at home. The findings suggest that iCT-SAD might be a promising option for Hikikomori clients who have social anxiety problems, within the recommended stepped-intervention approach.

## 1 Introduction

Hikikomori (prolonged social withdrawal), a cultural expression of distress included in the 5th text revision of the Diagnostic and Statistical Manual of Mental Disorders (DSM-5-TR) (1), was first clearly defined in 1990 in Japan. However, it has since been discussed as a hidden worldwide epidemic and a significant social and healthcare issue (2–4). In Japan, 1.46 million people are considered to be Hikikomori, and about 30% of these experience comorbid anxiety disorders (5–7). The World Mental Health Japan Survey also reported that 35% of Hikikomori individuals had pre-existing psychiatric disorders, with social anxiety disorder (SAD) representing the most common condition (8). To date, most research on Hikikomori has been focused on epidemiology and phenomenology rather than intervention/treatment. Although some studies exist suggesting the effectiveness of family-support interventions (9, 10), little is known about psychotherapeutic approaches for individuals with Hikikomori (11).

Here, in accordance with the Case Report (CARE) guidelines (12), we present a case of Hikikomori wherein a therapist-guided internet-delivered cognitive therapy for social anxiety disorder (iCT-SAD) (13–15) was effective in improving social anxiety symptoms and social interaction behaviors.

## 2 Case presentation

Akira (a pseudonym), a 30-year-old single male with Hikikomori, lived at home with his parents and had been self-isolating for six years. At assessment, he reported a number of social concerns, including difficulties establishing relationships with others, being in groups of more than 4-5 people, and talking to people his own age. He described a fear of sweating and worried that others would think he was weird or odd. He was concerned about being unemployed but was anxious about finding a new job. His parents were supportive, and there was no family conflict. Akira occasionally did leave the house to go for a walk, but he generally did this at night when there were fewer people around. In the year leading up to the intake interview with staff at the Hikikomori Community Support Center (just before the initial assessment with the therapist who provided iCT-SAD), he had not been able to interact with others, not even online.

As a child, Akira showed normal developmental milestones and did not experience social difficulties at school or university. After graduating, he worked as a part-time store clerk for a year and as a full-time event organizer for two years. He first experienced social anxiety symptoms at age 24, which he linked to harassment in the workplace, and he was eventually forced to leave his job. Since then, he has been unemployed, and socially isolated at home. He was formally diagnosed with SAD at age 29. He was prescribed medication but soon discontinued this because he felt there was no benefit. The present referral arose after Akira was offered a job but declined it due to his anxiety about not being able to do the job. His parents approached the Hikikomori Community Support Center, leading to Akira visiting the center for an interview, during which his need for psychological support was identified. However, Akira was anxious about attending standard weekly in-person therapy sessions, so it was suggested, and he agreed, that an online format of evidence-based psychological treatment could be a helpful way to overcome his problems (regarding both treatment and access). The first author (MS), a psychologist experienced in supporting individuals with Hikikomori and their families, provided the initial assessment and treatment.

At the initial assessment, conducted via videoconference, the therapist identified that Akira met the DSM-5 diagnostic criteria for SAD, which was further confirmed by a psychiatrist at the Hikikomori Community Support Center. The severity of social anxiety was assessed using the self-report version of the Liebowitz Social Anxiety Scale (LSAS) (16). His baseline LSAS score was 80, indicating a severe social anxiety problem (defined as over 80). The therapist also confirmed that Akira met the following diagnostic criteria for Hikikomori proposed by Kato et al. (17): a) marked social isolation in one’s home; b) duration of continuous social isolation of at least 6 months; and c) significant functional impairment or distress associated with the social isolation. The level of social interaction behaviors (a low level is a core characteristic of Hikikomori) was assessed using the Adaptive Behaviors Scale for Hikikomori-Self Report (ABS-H-SR) (18). A previous study reported that the mean ABS-H-SR score was 27.0 among Hikikomori individuals (and 52.1 among non-Hikikomori individuals) (18), and Akira’s baseline score was 23.

Akira had no current comorbid psychiatric disorders and was not taking psychotropic medication or receiving other psychological therapies. His baseline depression score on the Patient Health Questionnaire-9 (PHQ-9) (19) was 12, indicating a moderate level of depressive symptoms; however, his condition did not meet the DSM-5 diagnostic criteria for major depressive disorder. Akira believed that the workplace harassment he experienced at the age of 24 may have contributed to the onset of his social anxiety problems. Nevertheless, he did not experience intrusion symptoms or avoidance associated with this event, leading the therapist to rule out post-traumatic stress disorder.

## 3 Treatment course

### 3.1 About the treatment (iCT-SAD)

Akira received iCT-SAD, which is a therapist-guided internet-delivered program. See Stott et al., 2013 (13) for more details. iCT-SAD follows a modular structure and replicates the key treatment components of individual face-to-face cognitive therapy (CT); face-to-face CT is recommended as the gold standard psychological treatment in clinical practice guidelines in multiple countries (e.g. UK, Japan, Canada, Germany, and Australia and New Zealand) (20–24). The original English-language version of iCT-SAD has strong efficacy in the treatment of SAD through randomized controlled trials in the UK and Hong Kong (14, 25). Recently, a translated and culturally adapted version of iCT-SAD has been developed for Japanese clients (26), and its preliminary efficacy and acceptability for Japanese clients with SAD have been demonstrated in a pilot trial (15).

iCT-SAD consists of a 14-week treatment phase with a 3-month follow-up phase. Therapists support their clients via telephone calls, asynchronous and SMS messaging, and occasional video calls via webcam on the iCT-SAD website. Clients are encouraged to complete eight core modules in the first two weeks in order to promote initial engagement and motivation. Treatment is then tailored to the client’s individual concerns using a range of specific modules covering common fearful beliefs or problems.

Akira’s therapist received weekly individual supervision from the second author (NY), who is an expert in providing Japanese iCT-SAD but has no previous experience supporting Hikikomori clients. The third author (GRT) from the UK iCT-SAD team also provided bimonthly “supervision-of-supervision” to NY. All supervision was conducted online.

**Figure 1:**
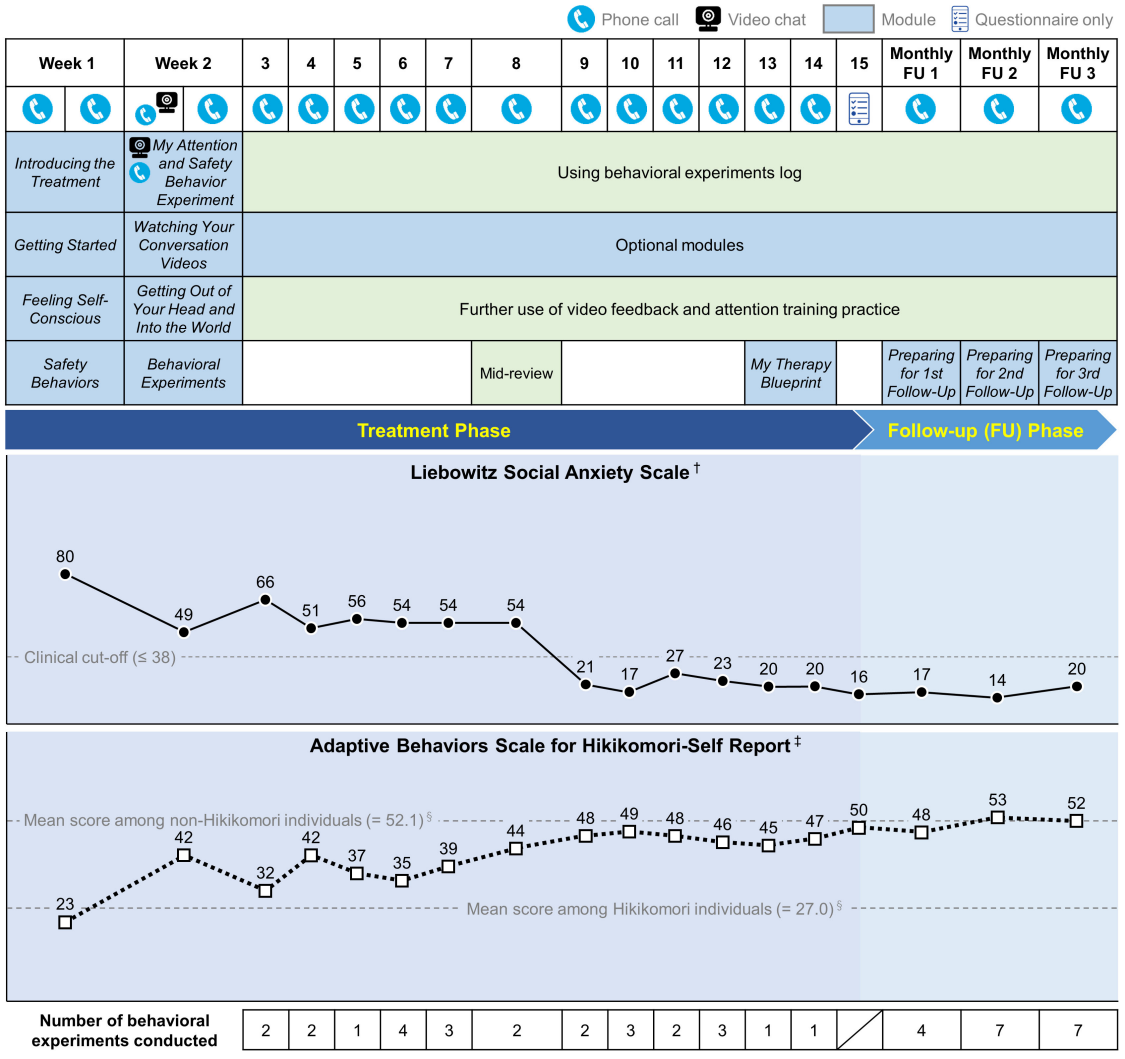
Overview of the treatment content and scores on social anxiety (Liebowitz Social Anxiety Scale) and social interaction behaviors (Adaptive Behaviors Scale for Hikikomori-Self Report) across treatment. ^†^ Lower scores indicate better outcome. ^‡^ Higher scores indicate better outcome. ^§^ Data from Nonaka & Sakai (2022) (18).

**Figure 1** presents an overview of the iCT-SAD treatment structure, together with Akira’s scores on social anxiety (LSAS) and social interaction behaviors (ABS-H-SR) throughout treatment.

### 3.2 Treatment phase (14 weeks)

In line with the standard iCT-SAD treatment protocol, during the first two weeks of treatment, two telephone calls per week were scheduled to support Akira’s initial engagement. He completed eight core modules covering key concepts of social anxiety and its maintenance: “*Introducing the Treatment*,” “*Getting Started*,” “*Feeling Self-Conscious*,” “*Safety Behaviors*,” “*My Attention and Safety Behavior Experiment*,” “*Watching Your Conversation Videos*,” “*Getting Out of Your Head and Into the World*,” and “*Behavioral Experiments*.” In the “*My Attention and Safety Behavior Experiment*” module, Akira worked with therapist support on a series of brief conversations with a stranger (a colleague of the therapist) via webcam. The interactions in this experiment were recorded, and Akira then reviewed these recordings under the guidance of the “*Watching Your Conversation Videos*” module. By reviewing recordings of their social interactions, he noticed discrepancies between his negative self-perceptions and his actual performance in the interaction, leading him to see that his self-image (distorted negative self-image) was incorrect. Through working on the “*Feeling Self-Conscious*” and “*Getting Out of Your Head and Into the World*” modules, Akira practiced systematic training in externally focused, non-evaluative attention both in non-social and social situations. Akira and his therapist also discussed how to apply his initial learning from treatment by taking walks outside during the daytime, including in crowded places (e.g. the largest shopping mall in his city). Scores on LSAS and ABS-H-SR substantially improved from Week 1 to Week 2.

During Weeks 3-14 of the remaining treatment phase, the therapist supported Akira through weekly phone calls. They worked together to plan numerous behavioral experiments using the relevant modules (e.g. “*Behavioral Experiments*” and “*Decatastrophizing*”) and the record sheets within the program, and he completed these experiments independently. These experiments tested his specific fearful concerns about social situations while focusing his attention externally and dropping safety behaviors. Behavioral experiments are a key treatment component of CT/iCT-SAD, and Akira was encouraged to conduct experiments regularly throughout the remaining treatment phase. Treatment was also tailored to his specific concerns using optional treatment modules, such as “*Sweating*,” “*Having Conversations*,” “*Managing My Inner Critic,” and “Leaving the Past Behind*.” Akira’s completed behavioral experiments included having brief conversations with store staff (e.g. convenience store, electronic store, and pet shop), making phone calls to stores, and going to a store while sweating. The therapist signposted him to a free online speech practice group, where several times he gave an impromptu speech.

Just before the end of the treatment phase, in the “*My Therapy Blueprint*” module, Akira summarized his achievements and major learning points thus far during the treatment phase, and planned how to apply and expand his learning in order to deal with any future setbacks (relapse prevention). Akira’s scores on LSAS and ABS-H-SR further improved, and after Week 9, LSAS scores remained below the clinical cut-off (≤ 38 points), with ABS-H-SR scores close to the mean of non-Hikikomori individuals (52.1 points) reported in a previous study (18). Additionally, his depression scores on the PHQ-9 exhibited improvement, registering at 4 points by Week 15, well below the clinical cut-off point of 10 (19).

### 3.3 Follow-up phase (3 months)

During the monthly follow-up phase, Akira continued to conduct behavioral experiments regularly and further expanded his social life. This included regularly attending vocational seminars at the Youth Career Support Center, and inviting an old friend to go out for dinner. Just before the 3-month follow-up, he had started looking for a new job with support from the Youth Career Support Center.

As seen in **Figure 1**, his final LSAS score was 20 points (80 at baseline [clinical cut-off: ≤ 38 points]), and for ABS-H-SR was 52 points (23 at baseline [the mean score among non-Hikikomori individuals: 52.1]). Based on the standardized definition of reliable response to treatment (LSAS improvement > 31%) and remission criteria for SAD (LSAS reduction ≥ 12 points, combined with an LSAS final score ≤ 38, and not meeting SAD diagnostic criteria) (13–15, 26), he was considered a treatment responder and remitted from SAD.

After the completion of iCT-SAD, the staff at the Hikikomori Community Support Center took over support for Akira. Almost two months after completing the follow-up phase, Akira eventually started working at an electronics store.

### 3.4 Therapist activity throughout the treatment and follow-up phase

Throughout the entire treatment (including the follow-up phase), the therapist conducted 18 phone calls with Akira, averaging 20.7 minutes per call. Additionally, one video call was made, lasting 94 minutes. The total duration of direct live communication with Akira during the treatment phase was 417 minutes, with an additional 49 minutes during the follow-up phase, bringing the total time to 7.8 hours (equal to 466 minutes). This therapist-client direct communication time is slightly less than the average time of 10.1 hours among Japanese clients with SAD in our pilot study (15). In addition to the direct live communication, the therapist also sent 52 asynchronous or SMS text messages to Akira, which is almost the same as the average of 48.8 messages sent to Japanese SAD clients in our pilot study (15).

### 3.5 Overall client’s treatment engagement and acceptability

Throughout the treatment, no adverse events or unanticipated events were observed. Akira attended all the scheduled phone call sessions with the therapist.

Metadata about the client’s activity on the iCT-SAD program was automatically collected/recorded. He completed all 25 core and optional modules released to him. Across the treatment, Akira logged into the program for 59 hours and 6 minutes, during which he worked on the modules for 31 hours and 27 minutes. He completed 44 behavioral experiments, which is higher than the average number of completed experiments (17.6) among Japanese clients with SAD in our pilot study (15). These results reflected Akira’s high level of treatment engagement and suggest good treatment acceptability.

### 3.6 Client feedback

At the end of the follow-up phase, Akira was invited to complete an online feedback survey about his experience with the treatment. This survey included questions about his experience with different modules, treatment components, and therapist behavior, which were rated on a Likert scale from 0 (not helpful at all) to 5 (extremely helpful).

The modules he rated as extremely helpful (5) in overcoming his social anxiety were “*My Attention and Safety Behavior Experiment,” “Behavioral Experiments,” and “Managing My Inner Critic*.” The iCT-SAD features that he perceived as very helpful (4) in overcoming his social anxiety were the messaging function (emails) on the website, SMS text messages, phone calls, and Webcam chats. The most helpful therapist behaviors were making suggestions for behavioral experiments, general encouragement, and clarification for completed modules and behavioral experiments, all of which were rated as 4. He also rated the amount of contact from his therapist on a Likert scale from 0 (Too little contact) to 5 (Too much contact); his rating was 3, indicating “Just the right amount.” Overall, he was very satisfied (4) with the treatment based on a Likert scale from 0 (not satisfied at all) to 5 (extremely satisfied).

## 4 Discussion

This case study demonstrates the principle that evidence-based psychological interventions focusing on social anxiety can be effective for clients with Hikikomori. This is important because SAD is the most common psychiatric disorder preceding the onset of Hikikomori (8), and a previous international survey revealed that many clients with Hikikomori seek treatment and prefer psychological treatment (especially individual format) over drug treatment (3).

Inspection of Akira’s weekly scores indicated a particular improvement on the LSAS between Weeks 8 and 9. This change meets the criteria to be considered a “sudden gain,” a phenomenon that has been shown to occur in 51% of participants undertaking iCT-SAD, and is associated with better overall outcomes (27, 28). Although we cannot determine the exact cause of the sudden gain in this instance, we note that Akira and his therapist were planning various behavioral experiments in the weeks leading up to this point, and he successfully attended the online speech practice group during Week 8 for the first time. This may highlight the value of signposting and supporting Hikikomori clients with SAD to engage with external group social activities of this type.

It is particularly worth noting that the positive feedback about the client’s experience with the treatment indicated that the online mode of treatment delivery, along with a variety of relevant modules, is acceptable and may facilitate clients’ engagement with treatment at home. It has been noted that online technology (e.g. online-based treatments/interventions, and smartphone apps) may be useful in supporting Hikikomori clients. For example, although not directly related to intervention, there were some Hikikomori clients who began to venture out of their homes to play the online game “Pokémon Go” which utilizes location information and augmented reality, and in which users need to go to different outside places to search for and catch Pokémon (29). This example may suggest the possibility of an online mode of treatment delivery, but caution is needed because a previous international survey reported that clients with Hikikomori were more likely to have an interest in an in-person treatment delivery format than in an online delivery format via webcam (3). Despite such caution, although iCT-SAD includes occasional video calls with therapists (e.g. the “*My Attention and Safety Behavior Experiment*” module), most client support is provided via telephone calls and text messaging. This feature of iCT-SAD may have contributed to the client’s positive experience with and acceptance of the treatment.

It should be noted that individual support alone is not sufficient to support individuals with Hikikomori, as it is a complex and multifaceted condition. The Japanese government guidelines (30, 31) recommend a four-step approach for Hikikomori: 1) family support and first contact with the individual and his/her evaluation; 2) individual support; 3) training in an intermediate-transient group situation; and 4) a social participation trial. Within this step-by-step approach, iCT-SAD might offer a promising option as the second step (i.e. individual support) for Hikikomori clients who have social anxiety problems. In addition to individual support, it is essential to provide careful support and education for family members, enhance their social skills and networks, develop the individual’s career skills, and offer employment support to improve their well-being and promote recovery.

To summarize, the findings from this case study suggest that iCT-SAD might be a promising option for Hikikomori clients who have social anxiety problems, within the four-step approach for Hikikomori recommended by the Japanese government guidelines (30, 31).

## Data availability statement

The original contributions presented in the study are included in the article/supplementary material. Further inquiries can be directed to the corresponding author.

## Ethics statement

Written informed consent was obtained from the individual for the publication of any potentially identifiable images or data included in this article.

## Author contributions

MS: Conceptualization, Investigation, Writing – original draft, Writing – review & editing, Funding acquisition. NY: Conceptualization, Supervision, Writing – original draft, Writing – review & editing, Funding acquisition. GT: Supervision, Writing – original draft, Writing – review & editing, Funding acquisition. DC: Conceptualization, Writing – original draft, Writing – review & editing, Funding acquisition.
